# From OPC to Oligodendrocyte: An Epigenetic Journey

**DOI:** 10.3390/cells8101236

**Published:** 2019-10-11

**Authors:** Assia Tiane, Melissa Schepers, Ben Rombaut, Raymond Hupperts, Jos Prickaerts, Niels Hellings, Daniel van den Hove, Tim Vanmierlo

**Affiliations:** 1 Department of Immunology, Biomedical Research Institute, Hasselt University, Hasselt 3500, Belgium; assia.tiane@uhasselt.be (A.T.); melissa.schepers@uhasselt.be (M.S.); ben.rombaut@uhasselt.be (B.R.); niels.hellings@uhasselt.be (N.H.); 2 Department of Neurology, Zuyderland Medical Center, Sittard-Geleen 6130 MB, The Netherlands; r.hupperts@zuyderland.nl; 3 Department Psychiatry and Neuropsychology, European Graduate School of Neuroscience, School for Mental Health and Neuroscience, Maastricht University, Maastricht 6200 MD, The Netherlands; jos.prickaerts@maastrichtuniversity.nl (J.P.); d.vandenhove@maastrichtuniversity.nl (D.v.d.H.); 4 Department of Psychiatry, Psychosomatics and Psychotherapy, University of Wuerzburg, Wuerzburg 97080, Germany

**Keywords:** oligodendrocyte, epigenetics, myelination

## Abstract

Oligodendrocytes provide metabolic and functional support to neuronal cells, rendering them key players in the functioning of the central nervous system. Oligodendrocytes need to be newly formed from a pool of oligodendrocyte precursor cells (OPCs). The differentiation of OPCs into mature and myelinating cells is a multistep process, tightly controlled by spatiotemporal activation and repression of specific growth and transcription factors. While oligodendrocyte turnover is rather slow under physiological conditions, a disruption in this balanced differentiation process, for example in case of a differentiation block, could have devastating consequences during ageing and in pathological conditions, such as multiple sclerosis. Over the recent years, increasing evidence has shown that epigenetic mechanisms, such as DNA methylation, histone modifications, and microRNAs, are major contributors to OPC differentiation. In this review, we discuss how these epigenetic mechanisms orchestrate and influence oligodendrocyte maturation. These insights are a crucial starting point for studies that aim to identify the contribution of epigenetics in demyelinating diseases and may thus provide new therapeutic targets to induce myelin repair in the long run.

## 1. Introduction

**Figure 1:**
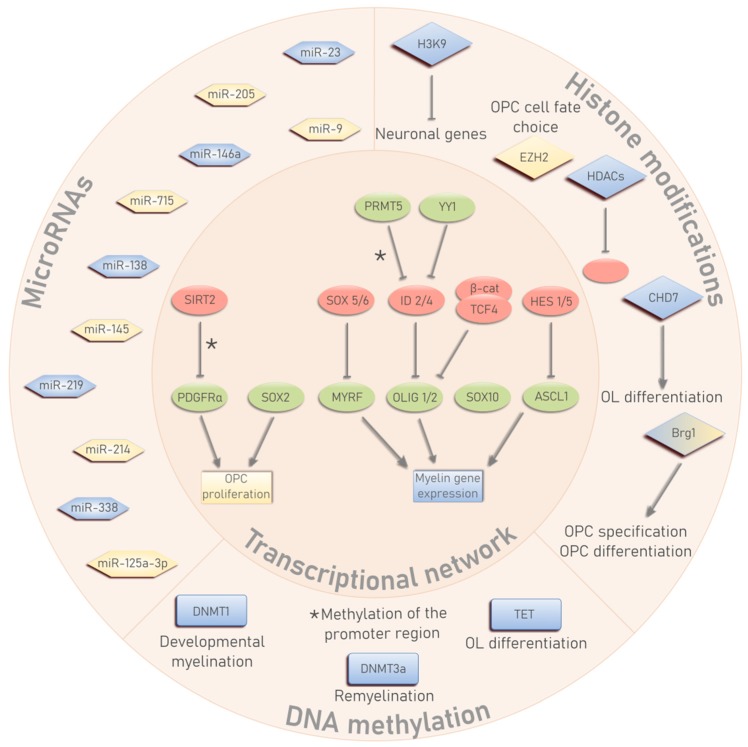
An overview of the transcriptional and epigenetic regulation of oligodendrocyte precursor cell (OPC) proliferation and oligodendrocyte (OL) development. Transcription factors that exert a positive or negative effect on these processes are depicted in green and red, respectively. Pro-proliferative factors are visualized in yellow, whereas pro-differentiation factors are blue. * Methylation of the promoter region.

Oligodendrocytes (OLs) are myelinating glial cells within the central nervous system (CNS) that insulate neuronal axons to provide them with trophic, metabolic and functional support. OLs are generated from oligodendrocyte precursor cells (OPCs) via a consecutive process of cell cycle exit, maturation, and differentiation [1]. OPCs arise during early development, persist throughout a lifetime and occupy around 5%–10% of the total number of cells in the brain [2,3]. In response to both intrinsic molecular cues and extracellular signals, OPCs are able to withdraw from their proliferative stage and differentiate into myelin-producing OLs [4]. Consequently, alterations in these extrinsic stimuli, such as an increase in inhibitory ECM molecules (LINGO, glycosaminoglycans, fibronectin) or secreted factors (BMP, FGF), hamper differentiation, possibly via an upstream effect on transcriptional and epigenetic processes that regulate OL differentiation [5]. Indeed, current evidence indicates that epigenetic mechanisms, comprising DNA methylation, histone modifications and microRNAs (miRNAs), play an essential role in the regulation of OL lineage development. As such, epigenetic signatures translate extracellular signals into functional cellular changes and coordinate the transcriptional machinery that is responsible for the differentiation process [6,7]. This review provides an overview of the current understanding of the physiological process of OL lineage development and how the different epigenetic mechanisms are involved in the regulation of this process (Figure 1). Furthermore, we discuss how this epigenetic fingerprinting is altered during ageing and in neurological conditions.

## 2. OL Differentiation and the Transcriptional Network

OPCs arise from the ventricular zone during early development, proliferate and migrate their way into the different developing areas of the brain, where they differentiate into myelin-forming OLs [8]. Unlike most progenitor cells, OPCs persist throughout life as adult, self-renewing OPCs that can differentiate into newly formed myelinating OLs to maintain myelin plasticity or in response to damaging signals [9]. The differentiation of OPC into mature and myelin-producing OLs is a gradual and well-defined process that can be divided into four successive stages: proliferative OPCs, pre-OLs, differentiated OLs and myelinating OLs [10]. This process of OL differentiation, both during early development and in adult stages, is controlled by the combination of OL-specific transcription factors, extracellular signals, epigenetic modifications and signaling pathways. It is necessary to maintain a homeostatic balance between these molecular cues to allow for proper differentiation.

The regulatory network of transcription factors that controls OL lineage development has been extensively studied over the past decades [9,11,12]. These transcription factors regulate OPC proliferation, migration and differentiation and at the same time serve as stage-specific cell identity markers of the OL lineage [11]. In general, a distinction can be made between positive regulators, which boost and stimulate OL differentiation, and negative regulators, which function as inhibitory transcription factors for myelin genes and keep OPCs in a proliferative and non-differentiated state.

The main transcription factors that regulate OL lineage progression belong to the helix-loop-helix (HLH) family, such as the oligodendrocyte transcription factors (OLIG), hairy and enhancer-of-split homologs (HES) and inhibitor or differentiation (ID) proteins. OLIG2 is considered as one of the major and indispensable transcription factors during different stages of OL development. It is an essential factor during OPC specification, enhances OPC migration during early development, but also functions as a promoting factor of OL differentiation and regeneration in the adult life [13,14,15]. In contrast to OLIG2, the closely related OLIG1 is not directly involved during early brain development, but rather promotes OL differentiation and myelination after injury [16,17]. The achaete-scute homolog 1 (ASCL1 or MASH1) is another member of the HLH family that promotes early OPC specification and OL development [18]. Although it was considered to be mainly involved in early oligodendrogenesis, ASCL1 is also shown to be important during adult OL regeneration and remyelination [19]. In contrast, HES proteins, such as HES1 and HES5, function as differentiation inhibitors either by recruiting other repressor proteins to myelin gene promoters, or by inhibiting ASCL1 [12]. Similarly, the ID HLH transcription factors ID2 and ID4 inhibit OPC differentiation by binding to other members of the HLH family (OLIG1/2, ASCL1) and preventing their translocation from the cytoplasm to the nucleus [20,21].

Another family of transcriptional regulators are HMG-domain transcription factors, that are classified as the sex determining region Y-box (SOX) family, of which SOX10 is a well-established regulator involved in terminal OL differentiation and myelination, through its direct binding to the promoter region of myelin genes to enhance their [22,23]. Interestingly, SOX10 is expressed in all stages of the OL lineage and can thus serve as a general marker for OPCs/OLs [24]. In contrast, SOX5 and SOX6 inhibit OL differentiation by competing with SOX10 binding sites, thereby antagonizing its function [25]. SOX2 on the other hand, maintains OPCs in a proliferative and undifferentiated stage, but is indispensable for OPC expansion and OL regeneration during CNS remyelination [26,27]. Transcription factor 4 (TCF4, also known as TCFL2) is another important HMG-domain transcription factor and is a downstream effector of the Wnt signaling pathway. Through its binding to β-catenin, TCF4 acts as an inhibitor of myelin gene expression and impairs (re)myelination [28].

An additional class of OL-related transcription factors are zinc finger proteins (ZFP). Yin Yang 1 (YY1) stimulates OL differentiation by silencing inhibitor proteins, such as ID4 and TCF4 [29]. Other ZFPs that enhance OL maturation and differentiation are ZFP191, ZFP488 and the Smad interacting protein 1 (SIP1) [30,31,32,33]. Myelin regulatory factor (MYRF) was only recently discovered as a crucial regulator of CNS myelination [34]. MYRF is exclusively expressed in post-mitotic cells of the OL lineage, which signifies its essential role during terminal differentiation. The synergistic effect of MYRF and SOX10 leads to myelin gene activation and drives CNS myelination [23,34].

All the transcriptional regulators influence OL differentiation mainly by controlling the expression of genes that encode for the essential myelin-associated proteins, such as the myelin basic protein (MBP), proteolipid protein (PLP) and myelin-associated glycoprotein (MAG) [35,36]. The transcription factors either enhance or inhibit the expression of these myelin genes by directly binding to their promoter region, which eventually results in a spatiotemporal expression of myelin genes during the process of OL lineage development [37].

## 3. The Epigenetic Triumvirate in OL Development

OL lineage development and the regulation of the associated transcriptional program is highly influenced by various epigenetic processes. Epigenetic mechanisms are defined as modifications that affect gene expression without altering the DNA sequence itself and are heritable from mother to daughter cell [38,39]. Epigenetic control of gene expression is sustained via DNA methylation, modifications at histone tails of chromatin, and miRNAs. The interplay between these different modifications changes the physiological form of the DNA, thereby influencing the accessibility of specific transcription factors to their target regions in the genome [39,40]. In the following part of this review, we discuss how the different levels of epigenetic regulation influence OL differentiation and CNS myelination. 

### 3.1. DNA Methylation

DNA methylation, in particular CG methylation, is one of the most studied and long-lasting epigenetic modifications. CG methylation involves the addition of a methyl-group (–CH3) to a cytosine base followed by a guanine nucleotide, referred to as 5′cytosine–guanine–3′ dinucleotide (CpG) site. Although various definitions exist, so-called ‘CpG islands’ cover regions of more than 300 bp with a C/G-content of 50% at minimum and are mostly found within the promoters of protein coding genes [41]. Methylation of these CpG islands is generally associated with gene silencing due to the inability of transcription factors to bind to the methylated promoter region or via an additional recruitment of other repressor proteins [42,43]. DNA methylation is established by DNA methyltransferases (DNMTs) that add a methyl-group to cytosine (5mC). There are two distinct forms of DNMTs, DNMT1 and DNMT3a/b, which either maintain DNA methylation during replication or induce de novo methylation, respectively [44,45]. Contrarily, DNA methylation can be removed via gradual degradation of 5mC by the ten-eleven translocation (TET) enzymes [46,47], although DNMTs may serve the same purpose under certain conditions [48,49]. Hydroxylation of 5mC into hydroxy-methylated cytosine (5hmC) is the first step of the demethylation process. Interestingly, 5hmC patterns have shown to be abundantly present in the CNS of mammals [47,50]. 5hmC was first identified as an intermediate epigenetic mark during active DNA demethylation but has also been shown to represent a potentially independent and functionally distinct epigenetic marker in the brain [51,52].

One of the first studies that linked DNA methylation to OL development showed that neonatal rats treated with the DNMT-inhibitor 5-azacytidine (5-aza), displayed disrupted gliogenesis, concomitant with hypomyelination of the 11-day-old optic nerve. Postnatal inhibition of DNA methylation resulted in a reduced number of oligodendrocytes, whilst the number of astrocytes was less affected, indicating a higher vulnerability of OPCs to changes in DNA methylation [53]. Likewise, ablation of the *Dnmt1* gene in embryonic progenitor cells led to OPC growth arrest and resulted in severe hypomyelination. Moreover, this loss of *Dnmt1* seemed to alter splicing events, such as exon skipping and intron retention, in genes related to myelination, lipid metabolism and the cell cycle, indicating a crucial role of DNA methylation in relation to alternative splicing during neonatal OL development [54]. Although DNMT1 seemed to be an important regulator during developmental myelination, it seems to play a less prominent role during remyelination of the adult CNS [55]. After lysolecithin-induced demyelination of adult murine spinal cord white matter, higher levels of DNA methylation in differentiating OLs are accompanied by an increased expression of DNMT3a. Transgenic mice that lack *Dnmt3a* showed impaired OL differentiation and a reduced ability to remyelinate affected axons after injury [55]. Together, these studies suggest that maintenance of DNA methylation is important to ensure proper gliogenesis during developmental myelination, whilst de novo methylation is needed for the differentiation of adult OPCs into remyelinating OLs. On the opposite side of the methylation spectrum, TET enzymes also strongly influence OL differentiation [56]. Even though the three TET enzymes show different subcellular localization and unique expression patterns, they all seem to be equally important during OL development. Interestingly, knock-down of the *Tet* mRNA levels was associated with increased expression of HLH inhibitory transcription factors, such as ID2 and HES5, leading to suppression of myelin gene expression [56]. It however remains unclear whether TET enzymes directly inhibit the expression of these genes or whether the observed transcriptional change is mediated in an indirect manner. In general, epigenome-wide studies of stage-specific cells are still needed to unravel how and which exact CpG sites or islands change in their methylation status during OL lineage progression.

In relation to the transcriptional regulatory network of OL development, it has been shown that DNA methylation can regulate the temporal expression of these transcription factors. In a study of Huang et al., PRMT5 was identified as a pro-differentiation factor that binds to CpG-rich islands within the ID2 and ID4 genes. Subsequent DNA methylation of these regions led to silencing of the transcriptional inhibitors and resulted in OL differentiation [57]. In a similar fashion, SIRT2 was shown to translocate to the nucleus, inducing DNA methylation in the platelet-derived growth factor receptor α (PDGFRα) promoter region and initiating glial differentiation [58]. Interestingly, both PRMT5 and SIRT2 are classified as histone-modification enzymes, yet they are also known to induce epigenetic changes at the level of DNA methylation, thereby emphasizing the intricate relationship between different epigenetic mechanisms.

### 3.2. Histone Modifications

Histone modifications encompass a wide range of post-translational changes on histone tails, such as histone (de)acetylation, methylation, ubiquitination, and phosphorylation. These modifications can act separately or together to orchestrate chromatin dynamics and structure. Depending on the obtained histone code, DNA accessibility for polymerases and transcription factors can be either promoted or hampered [59].

The most prevalent type of histone modifications is (de)acetylation of the lysine (K) residues. Acetylation is established by histone acetyltransferases (HATs), whilst removal of the acetyl groups is maintained by histone deacetylases (HDACs). Histone acetylation neutralizes the positive charge of the lysine residues, resulting in a weaker interaction between the histone proteins and the DNA, eventually leading to an ‘open’ chromatin structure. Consequently, HDACs function to make the chromatin more compact, thereby preventing transcriptional processes from occurring [59,60]. Whereas not that many studies have directly assessed the role of HATs in OL development, HDACs have been shown to be heavily involved in different aspects of this process. In general, pharmacological inhibition of HDACs is associated with a decrease in OL maturation and differentiation, suggesting a crucial role of HDACs during OL development [61,62,63,64]. Treatment of OL in vitro cultures with the HDAC inhibitor trichostatin A (TSA), prevented the suppression of inhibitory transcription factors, such as ID2 and SOX11, in rats [63], and ID4, SOX2, and TCF4 in humans [64]. These data indicate that HDAC-mediated repression of genes that keep OPCs in a proliferative and undifferentiated state is necessary for the early onset of OL lineage progression. Indeed, it has been shown that HDAC functionality is restricted to a specific temporal window, as HDAC inhibitors seem to only suppress myelination during the early phase of OPC differentiation, but not after onset of myelination [62]. These observations are in line with recent findings, which show that HDACs are predominantly expressed in early OPC stages, compared to other stages of OL differentiation [65].

Interestingly, HDACs can also regulate and promote OL development in a (partly) histone-independent manner, as interaction of HDACs with other transcriptional regulators can result in repressive complexes that counteract the expression of OPC differentiation inhibitors. For instance, studies conducted on murine OPCs have shown that the pro-differentiation factor YY1 is recruited via HDAC1 to the promoter region of *Id2*, *Id4* and *Hes5*, where it can block the expression of these genes [66]. Protein deacetylation of OLIG1 by HDACs prevents its physical interaction with the inhibitory ID2 protein, stimulates its nuclear transportation and promotes OPC differentiation [67]. Furthermore, HDAC1/2 interact with TCF4 and antagonize its binding to β-catenin, thereby preventing its downstream function as an inhibitor of myelin gene expression [28].

Another type of histone modification that has been associated with OL development is histone methylation. Histone methylation can occur either on lysine or arginine side chains and is associated with both activation and repression of transcription, depending on the site of methylation [60]. During OL differentiation, the activity of the Histone H3 Lysine 9 (H3K9) methylation enzyme increases. This is accompanied by an increase of the associated repressive H3K9me3 mark at genes that regulate neuronal lineage development [68]. Furthermore, the catalytic subunit (EZH2) of the polycomb repressive complex (PRC) that is responsible for trimethylation of histone 3 (H3K27me3), promotes OPC cell fate choice from progenitor cells and stimulates OPC proliferation [69,70]. A decrease in histone H4R5 methylation via pharmacological inhibition or genetic ablation of PRMT5 results in poor OL differentiation and hypomyelination [71]. Likewise, deletion of PRMT1 leads to severe hypomyelination due to impaired OL maturation and disturbed myelin gene expression in OLIG2-positive cells [72].

Next to the abovementioned histone-modifying enzymes, ATP-dependent chromatin remodeling complexes have also been recently shown to influence and orchestrate OPC differentiation. These complexes make use of ATP as an energy source to reposition nucleosomes, thereby altering histone accessibility and gene transcription [73]. The helicase component of the SWI/SNF-related chromatin remodeling complex brahma-related 1 (Brg1, also known as Smarca4) is highly expressed in OPCs and is an essential factor during OPC specification and at the onset of OL differentiation. BRG1 interacts with the *Olig2* promoter in order to regulate its expression during early development [74]. As a positive feedback loop, BRG1 is consequently recruited by OLIG2 to enhance the expression of OL-associated genes [75]. One of these targets of BRG1 and OLIG2 is *Cdh7*, an ATP-dependent chromatin remodeler of the chromodomain helicase DNA-binding (CHD) family. CHD7 is highly expressed in differentiating OLs, and functions synergistically with SOX10 to enhance myelin-associated gene expression. Furthermore, CHD7 promotes the expression of other positive transcription factors during OL maturations, such as *Myrf* and *Olig1* [76]. Interestingly, deletion of either ATP-dependent remodeler (BRG1 or CHD7) resulted in a dysmyelinating phenotype in mice, suggesting that even though they have different targets and influence OL development at distinct stages, both BRG1 and CHD7 are indispensable factors during OL development and myelination [75,76].

### 3.3. MicroRNAs

Small non-coding RNAs (ncRNAs) are powerful endogenous regulators of gene expression. Many ncRNAs have been comprehensively described, such as Piwi-interacting RNAs (piRNAs), small interfering RNAs (siRNAs) and miRNAs, with these latter being the most widespread and abundant ncRNAs [77]. MiRNAs are small ncRNA molecules with an average length of 21–25 nucleotides and are most often transcribed from non-coding and coding protein introns [78]. By means of base-pair complementarity, a mature miRNA binds the seed-sequence at the 3′ untranslated region (3′UTR) of the target mRNA and subsequently negatively regulates its translation by repressing or degrading the mRNA [79,80,81]. Nevertheless, base-pair complementarity between miRNA and target RNA can sometimes be incomplete so that a single miRNA can target multiple 3′ UTR sequencing, leading to a cumulative reduction of gene expression that may orchestrate a common molecular pathway such as cell proliferation, development and differentiation [82].

During OL development, a coordinated interplay between multiple miRNAs determines OPC cell fate by downregulating intrinsic and extrinsic transcription factor expression [83,84]. The importance of miRNA-mediated gene repression in OPC differentiation is highlighted in animals lacking the DICER1 enzyme, which is an essential enzyme responsible for processing pre-microRNA (pre-miRNA) thereby forming mature miRNA. DICER1 mutant mice display a lack of mature miRNAs which is featured by a disrupted CNS myelination pattern due to the lack of differentiated OPCs [85,86]. MicroRNAome studies revealed a 10–100-fold induction of miR-219, miR-338 and miR-138 during OL differentiation [85,86]. Since direct targets of miR-219 include genes essential for maintaining OPC proliferation (e.g., *Sox6*, *Hes5* and *Pdgfrα*), its increase stimulates OPCs to exit from the proliferative cycle and enter differentiation [85]. By suppressing *Hes5* and *Sox6*, miR-219 indirectly elevates the expression of monocarboxylate transporters, leading to increased OL numbers and enhanced protein levels of MBP and CNP, which subsequently attenuates cuprizone-induced demyelination [87]. MiR-219 is additionally important for metabolic regulation of lipid formation and maintenance during OL maturation, rendering miR-219 essential in both early and late stages of OL differentiation [86]. MiR-219 cooperates synergistically with miRNA-138, which is essential for reaching the immature phase of OL differentiation, to regulate CNS myelination. Boosting the expression of solely these two miRNAs is sufficient to induce OL differentiation in vitro [88,89]. Furthermore, differentiation of human endometrial-derived stromal cells towards OLs is stimulated when miR-338 is overexpressed, emphasizing the importance of this miRNA in the regulation of OPC differentiation [90,91].

In contrast to the induction of several miRNAs, miR-9 is downregulated during OL differentiation [92,93]. In line with this, depleting miR-9 in OPCs stimulates OL differentiation, presumably through an increase in peripheral myelin protein 22 (PMP22) and serum response factor (srf) transcripts [92,94]. During OL differentiation, a comparable expression pattern of the developmentally regulated miR-125a-3p is observed. Oligodendroglial differentiation and maturation is impaired upon miR-125a-3p overexpression, which can be attributed to a decreased expression of genes involved in the differentiation process (e.g., GTPase RhoA, Neuregulin and p38) [95,96,97,98]. On the contrary, antago-miR treatment that inhibits miR-125-3p expression and subsequently stimulates OL differentiation, indicates the importance of miR-125a-3p suppression during oligodendroglial maturation [95].

Many other miRNAs have been described to be either positively or negatively involved in OL differentiation processes. In vivo studies have shown an increased generation of myelin proteins upon miR-146a overexpression in primary OPCs following demyelinating injuries, thereby highlighting the positive relationship between miR-146a and OL differentiation [99,100]. Similarly, miR-23 promotes CNS myelination via the suppression of lamin B1, which is a negative regulator of OL differentiation [101]. On the other hand, many miRNAs inhibit OL differentiation and therefore need to be downregulated during the transition of OPCs to OLs. The translation of essential proteins of the CNS myelin, such as myelin-associated oligodendrocyte basic protein (MOBP), claudin11/O4 and MBP, is suppressed by miR-214 [102,103], miR-205 [102] and miR-715 [97], respectively. Moreover, miR-145 has been shown to pair to its seeding sequence located in the 3′UTR of the gene coding for Myrf and consequently inhibits OPC differentiation [103,104]. Therefore, downregulating miR-214, miR-205, miR-715 and miR-145 is sufficient for the differentiation of OPCs into mature OLs. In contrast to regulating OL differentiation, at least one miRNA cluster, miR-17-92, has been shown to be involved in OPC expansion by targeting, among others, PTEN, and therefore regulating OL numbers both in vitro and in vivo [89,105]. Taken together, miRNAs have been shown to be critically involved in different steps of the process of OL development. Data have demonstrated that miRNA expression is dynamically and precisely regulated to control cellular differentiation, which offers new avenues for further therapeutic target identification for myelin-related pathologies.

## 4. Implications in Ageing and CNS Myelin Disorders

Current knowledge about the strong involvement of epigenetic mechanisms in OL development has led to new perspectives on OL- and myelin-related pathologies. Over the past years, a considerable amount of research has been conducted with regard to aberrant epigenetic regulation and its impact on OL regeneration and myelin repair. Hence, in this part of the review, we focus on what is known about epigenetic malfunctioning during OL regeneration and remyelination, both in the context of ageing and myelin-related pathologies.

### 4.1. Ageing

It is generally known that regenerative processes become less efficient with increasing age. A classic example is age-related deficits in remyelination, a process which is entirely dependent on OL regeneration to restore the myelin sheath [106,107,108]. The age-associated decrease in remyelination efficiency is attributed to a reduced level of OPC recruitment. Moreover, recruited OPCs show an impaired ability to differentiate into remyelinating OLs [107]. The relationship between ageing and epigenetic alterations has already been proposed before [109,110,111] and provides an incentive to link age-associated remyelination failure to changes in the epigenome of aged OPCs or OLs.

Up to now, only one study has connected changes in methylation in OPCs/OLs to cellular ageing [112]. Rat OPCs from the spinal cord showed an age-dependent decrease in methylation levels. Interestingly, no changes regarding TET activity or expression were observed. The global hypomethylation in aged OPCs rather correlated with a reduced expression and activity of DNMTs, and in particular DNMT1 [112]. Regarding histone modifications, mature OLs from the corpus callosum of older animals show increased levels of histone acetylation and a decreased rate of histone methylation, compared to younger mice. These histone changes were correlated with re-expression of inhibitory HLH-transcription factors, such as HES5 and ID4 [113]. As mentioned before, HDAC recruitment to these promoter regions is crucial for OPC differentiation and myelin formation. OPCs in demyelinated regions of older mice, however, fail in the recruitment of HDACs, resulting in the accumulation of transcriptional inhibitors and poor remyelination [114].

In a study conducted by Pusic et al., aged rats were exposed to a youthful environment in a Marlau-style enrichment cage to assess the effect on remyelination capacity [115,116]. Environmental enrichment promoted remyelination in aged rats, to a level comparable to younger animals. Interestingly, they found that serum-derived exosomes from both young and environmentally enriched stimulated rats displayed increased levels of miR-219, which is known to inhibit the expression of inhibitory myelin gene regulators and therefore promotes OL differentiation [115]. Exosomal delivery of such miRNAs could therefore be regarded as a potential therapeutic strategy to boost remyelination both in young and aged individuals.

### 4.2. Multiple Sclerosis

Multiple sclerosis (MS) is a multi-faceted immune-driven demyelinating disease of the CNS. MS is characterized by inflammation-induced demyelination during the early stages, which eventually results in gradual neurological disability as the disease progresses [117,118]. The concordance rate of identical twins to develop MS averages between 6%–30%, suggesting that the disease is only partially driven by genetic polymorphisms, but is largely attributed to environmental stimuli [119]. An increasing body of evidence suggests a role of epigenetically regulated mechanisms in the pathophysiology of MS. Numerous links have been made between environmental risk factors for MS and epigenetic changes [120,121,122]. Yet, most studies concerning epigenetics in MS are focused on the early, inflammatory stage of the disease [123,124,125]. Another important aspect of the disease is the subsequent endogenous repair process underlying remyelination of axons in order to cope with inflammatory damage. In the chronic stages of MS, however, these repair processes are hampered due to a differentiation block in OPCs [126,127]. New regenerative therapies, such as Opicinimab (anti-LINGO), are currently tested for their potential to boost remyelination in lesions that still contain undifferentiated OPCs [128]. Interestingly, even though the influence of epigenetics in progressive MS pathology is not clear yet, emerging data suggest an existing role in OL differentiation and maturation.

Analysis of MS postmortem samples revealed increased levels of MBP citrullination, a post-translational modification which renders the MBP protein less stable, leads to the degradation of myelin and can eventually result in the development of an auto-immune response against myelin [129,130]. MBP citrullination is carried out by the peptidyl arginine deiminase type-2 (PAD2) enzyme. Interestingly, the promoter region of the *PAD2* gene is hypomethylated in normal appearing white matter (NAWM) of MS patients, compared to control samples [130]. This implies that *PAD2* hypomethylation leads to a higher expression of the enzyme, which finally results in the destabilization and degradation of the myelin sheath in MS white matter. *PAD2* hypomethylation is, surprisingly, not brain-specific but can also be observed in peripheral blood mononuclear cells (PBMCs) of MS patients [131]. In a similar fashion, cell-free DNA (cfDNA) in peripheral blood samples of MS patients with an active disease course showed hypomethylated patterns of the *MOG* gene, which is associated with OL cell death and demyelinating events in the brain [132]. The correlation of methylation patterns between the brain and blood has gained interest over the past years for its potential application as a biomarker for neurodegenerative diseases [133,134,135], and could therefore also be used to monitor disease progression in MS. 

An epigenome-wide DNA methylation study (EWAS) was conducted on MS NAWM postmortem samples. Genes responsible for OL survival (*BCL2L2*, *NDRG1*) and myelination (*MBP*, *SOX8*) were hypermethylated and decreased in expression in MS-affected tissue, compared to controls [136]. While representing a valuable study, it is important to note that no distinction has been made between regular cytosine methylation and 5-hydroxymethylation (5hmC). Considering the functional consequences of 5hmC, but also to prevent underrepresentation of methylated cytosine values, 5hmC analysis should be taken along in CNS EWAS studies.

Another study that analyzed postmortem brain tissue of MS patients showed higher levels of histone acetylation in oligodendrocytes within chronic MS lesions, compared to non-neurological controls. These changes are associated with elevated HAT transcript levels and higher expression of inhibitory regulators (*TCF7L2*, *ID2*, *SOX2*). In contrast, OLs present in early MS lesions show the presence of deacetylated histones [137]. Since histone acetylation impairs OL differentiation and remyelination, these data could partially explain the poor remyelination capacity associated with progressive MS patients.

MiRNA analysis of brain samples of progressive MS patients showed upregulated levels of different miRNAs (miR-155, miR-338, miR-491), which target enzymes that are involved in the production of neurosteroids [138]. Opposing results were obtained from another study, in which they show that these miRNAs are downregulated in chronic, inactive MS lesions, compared to control white matter samples [139]. The discrepancy between these studies could be attributed to differences in the analyzed tissue, their control sample selection or the method of miRNA analysis, which makes it difficult to directly compare them to each other. Interestingly, the most significant downregulated hit from the latter study is miR-219, which, together with miR-338, is essential for OPC cell cycle exit and differentiation into myelin-producing OLs [85,88,91]. The absence of these miRNAs could thus underlie the differentiation block of OPCs in chronic demyelinated lesion of progressive MS patients. Moreover, miR-219 expression is also decreased in the cerebrospinal fluid (CSF) of MS patients, rendering it a possible biomarker for MS diagnosis [140].

It is however noteworthy that most of the abovementioned studies have been conducted on bulk tissue, leading to a possible noise introduced by the cellular heterogeneity. Since the observed epigenetic changes could be strongly influenced by cellular variation or cell numbers, cell type-specific validation is recommended to circumvent such bias [141,142].

### 4.3. Other Diseases with Myelopathy

Even though MS is regarded as the most common myelopathy of the CNS, many other neurological diseases are characterized by oligodendroglial injury and myelin disruption. Here, we briefly discuss how epigenetic changes impact OL regeneration and remyelination in relation to these other demyelinating diseases.

Ischemic stroke, caused by a cerebral artery occlusion, is an important cause of death worldwide and the majority of survivors often struggle from severe neurological disabilities throughout the lifespan. Molecularly, ischemic stroke can be characterized by a disrupted architecture of neuronal synapses, neuronal loss and loss of glial cells, including oligodendrocytes, leading to prominent white matter demyelination [143]. During stroke recovery, endogenous repair processes are initiated and include axonal growth, synaptic plasticity, angiogenesis, neurogenesis, and oligodendrogenesis. Interestingly, during early brain recovery following ischemic stroke, HDAC1 and HDAC2 levels were shown to be increased in white matter OPCs at the peri-infarct region [144,145]. Mature OLs showed a retained increase of HDAC2 following stroke, while HDAC1 levels were decreased, indicating that individual HDACs family members play distinct roles during recovery after stroke [144]. In line, pan-HDAC inhibitors have repeatedly shown to protect OLs from ischemia-induced cell death and subsequently increase oligodendrogenesis [146,147,148]. However, contradictory results have been observed for the pan HDAC inhibitor suberoylanilide hydroxamic acid (SAHA) as its treatment suppressed OPC survival, leading to detrimental effects for the myelinating brain during stroke recovery [149]. Interestingly, not only HDAC modifications have shown their importance during oligodendrogenesis following stroke, but also miRNAs have been widely investigated for their therapeutic and diagnostic properties [150]. In ischemic white matter regions, miR-9 and miR-200b levels were decreased, concomitant with an increased differentiation state of OL lineage cells [94,151]. However, the majority of the investigated miRNAs showed an increased expression pattern following stroke. For example, rodent models for ischemic stroke showed a high presence of miR-146a, miR-138, miR-338, miR-423-5p, miR-200b, miR-298, miR-205, miR-107 and miR-145 [99,152,153,154], all of which have a negative impact on OPC proliferation, which is actually necessary in the early phase after stroke injury to replenish the pool of lost OPCs. Interestingly, circulating miRNA levels have been measured in stroke patients to provide new therapeutic and minimally invasive diagnostic insights. Measuring miR-146a levels, for example, can segregate the acute phase from the subacute phase during ischemic stroke, thereby highlighting the usefulness of miRNAs for future stroke research [155].

X-linked adrenoleukodystrophy (X-ALD) is a genetic disorder caused by a mutation in the *ABCD1* gene and characterized by progressive demyelination of the CNS [156]. An important aspect of this disease is the absence of remyelination capacities, even after successful hematopoietic stem cell transplantation [157]. X-ALD patients endure progressive impairment of cognition, vision, hearing and motoric function, eventually leading to total disability [158]. An EWAS, conducted on white matter samples of the prefrontal cortex of X-ALD patients, revealed differential DNA methylation in genes involved in OL differentiation. Myelin genes, such as *MBP*, *PLP1*, *MOG* and *CNP* were hypermethylated in X-ALD patients compared to age-matched controls. Furthermore, transcriptional inhibitors (*ID4* and *SOX2*) displayed an increased expression in these patients, suggesting a disturbed HDAC activity [157]. In line with this, treatment with SAHA prevented OL cell loss both in vitro and in vivo by counteracting the very long chain fatty acid (VLCFA) derangement associated with X-ALD pathology [159]. Another type of leukodystrophy, adult-onset autosomal dominant leukodystrophy (ADLD) is characterized by duplication of the gene that codes for lamin B1 (*LMNB1*), which leads to overexpression of LMNB1 and causes severe myelin loss [160]. Interestingly, miR-23 has been identified as a negative regulator of lamin B by targeting its transcript levels and could therefore be considered as a therapeutic strategy for ADLD [161].

Schizophrenia has also been associated with OL dysfunction. Interestingly, the CpG island within the promoter region of *SOX10* is hypermethylated in brains of patients with schizophrenia, which is directly associated with a decreased expression of *SOX10* and other OL-related genes [162].

## 5. Therapeutic Perspectives: From Pharmaceuticals to (epi) Gene Therapy to IPSCs

It is clear that epigenetic modifications strongly influence OL development and functional remyelination in a wide variety of diseases. Targeting these epigenetic alterations could therefore be considered as a new therapeutic strategy to overcome remyelination failure. Most attempts to pharmacologically manipulate epigenetic modulations are based on the use of inhibitors of epigenetic enzymes, such as 5-aza, TSA and valproic acid (VPA) [163,164]. However, such pan-epigenetic inhibitors are non-specific due to their pleiotropic impact at a genome-wide level. Furthermore, these compounds are known to have low chemical stability and are cytotoxic at higher doses, which limits their potency to be used in a cellular microenvironment [165,166]. Recent improvements in the field of epigenetic editing have disclosed the use of DNA-binding proteins, such as zinc-finger proteins (ZFPs), transcription activator-like effectors (TALEs) and type II clustered regularly interspaced short palindromic repeat (CRISPR)/Cas9, as new synthetic epigenomic engineering tools [167,168,169,170]. These DNA-binding proteins are linked to epigenetic modifiers and serve to guide them to a specific region in the genome, thereby altering the epigenome at specific loci. Even though many advances have been made regarding these new epigenetic editing techniques, their applicability in the clinic may require, next to ethical considerations, additional research as their safety and efficacy remain to be disclosed. In particular, the off-target effects and undesired genomic binding of these DNA-binding proteins are still considered as one of the major hurdles for their therapeutic application [171]. 

Autologous cell-based therapies have emerged as a promising technique to restore OL dysfunction. Mature and fully differentiated OLs derived from induced pluripotent stem cells (iPSCs) have shown to successfully remyelinate axons in rodents [172]. Interestingly, human iPSC-derived OPCs show the same epigenetic signature during their differentiation process into mature OLs as seen in normal OL development [173]. Furthermore, generation of oligodendrocytes from progressive MS patient-derived iPSCs results in functional and myelinating cells, in contrast to the resident non-myelinating OPCs in the CNS [174]. Since the epigenetic signature of OPCs/OLs can be disturbed in a pathological context, reprogramming patient-derived iPSCs into OLs and repopulating lesion sites with these cells could be considered as a promising remyelinating strategy.

## 6. Concluding Remarks

In this review, we have discussed how different epigenetic modifications influence OL development and lineage progression and how this is dysregulated in demyelinating conditions. Epigenetic mechanisms function as a precise gateway control system that governs the transcriptional machinery in a spatiotemporal manner. In CNS demyelinating diseases, these epigenetic mechanisms are found to be altered, concomitant with increased levels of transcriptional inhibitors and resulting in a differentiation block of OPCs. Targeting these epigenetic processes, either by pan-inhibitors or via CRISPR/Cas9-mediated epigenetic editing, could therefore be a potential strategy to boost OL differentiation and (re)myelination. Taken together, epigenetic research has earned its place within the universe of OL development and further studies will contribute to the complete understanding of CNS myelin disorders.
